# 2-Phenyl­biguanidinium hydrogen succinate methanol monosolvate

**DOI:** 10.1107/S160053681004585X

**Published:** 2010-11-17

**Authors:** Irena Matulková, Ivana Císařová, Ivan Němec

**Affiliations:** a Department of Inorganic Chemistry, Faculty of Science, Charles University in Prague, Hlavova 2030, 128 40 Prague 2, Czech Republic; b Department of Spectroscopy, J. Heyrovský Institute of Physical Chemistry of the ASCR, v.v.i., Dolejškova 3, 182 23 Prague 8, Czech Republic; c Department of Inorganic Chemistry, Faculty of Science, Charles University in Prague, Hlavova 2030, 128 40 Prague 2, Czech Republic

## Abstract

In the crystal of the title compound, C_8_H_12_N_5_^+^·C_4_H_5_O_4_^−^·CH_3_OH, the hydrogen succinate anions form infinite [010] chains *via* short, almost symmetrical, O⋯H⋯O hydrogen bonds. The 2-phenyl­biguanidium cations inter­connect these chains into layers lying parallel to the *bc* plane by way of N—H⋯O links. These planes only weakly inter­act in the direction of the *a* axis *via* C—H⋯π contacts between offset phenyl rings, leaving as much as 17% of the unit-cell volume accessible for the solvent. However, the methanol solvent mol­ecules could not be resolved due to extensive disorder and their assumed presence was removed from the overall scattering by the *PLATON* SQUEEZE procedure.

## Related literature

Biguanides forms stable complexes, see: Marchi *et al.* (1999 ▶); Ray (1961 ▶); Anderson *et al.* (1995 ▶) and also have applications in medicine, see: Sirtori & Pasik (1994 ▶); Clement & Girreser (1999 ▶); Thompson *et al.* (1999 ▶); Ross *et al.* (2004 ▶); Woo *et al.* (1999 ▶); Watkins *et al.* (1987 ▶); Morain *et al.* (1994 ▶); Shapiro *et al.* (1959*a* ▶,*b* ▶). Ionic crystal structures containing biguanide cations are formed by relatively strong hydrogen bonds, see: Martin *et al.* (1996 ▶); Martin & Pinkerton (1996 ▶); Pinkerton *et al.* (1978 ▶); Matulková *et al.* (2008 ▶, 2010 ▶). For the SQUEEZE method used to solve the structure, see: van der Sluis & Spek (1990 ▶).
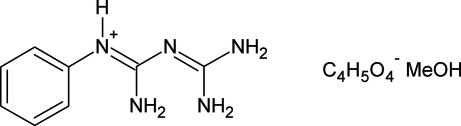


## Experimental

### 

#### Crystal data

C_8_H_12_N_5_^+^·C_4_H_5_O_4_^−^·CH_4_O
*M* *_r_* = 327.35
Monoclinic, 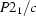

*a* = 10.3280 (3) Å
*b* = 6.4590 (1) Å
*c* = 24.6770 (6) Å
β = 94.0480 (13)°
*V* = 1642.06 (7) Å^3^
*Z* = 4
Mo *K*α radiation
μ = 0.10 mm^−1^
*T* = 293 K
0.45 × 0.4 × 0.18 mm

#### Data collection

Nonius KappaCCD diffractometer
18123 measured reflections
3568 independent reflections
2613 reflections with *I* > 2σ(*I*)
*R*_int_ = 0.030

#### Refinement

*R*[*F* ^2^ > 2σ(*F* ^2^)] = 0.056
*wR*(*F* ^2^) = 0.174
*S* = 1.07
3568 reflections
191 parameters
H-atom parameters constrained
Δρ_max_ = 0.25 e Å^−3^
Δρ_min_ = −0.23 e Å^−3^

### 

Data collection: *COLLECT* (Hooft, 1998 ▶) and *DENZO* (Otwinowski & Minor, 1997 ▶); cell refinement: *COLLECT* and *DENZO*; data reduction: *COLLECT* and *DENZO*; program(s) used to solve structure: *SIR92* (Altomare *et al.*, 1994 ▶); program(s) used to refine structure: *SHELXL97* (Sheldrick, 2008) ▶; molecular graphics: *PLATON* (Spek, 2009) ▶; software used to prepare material for publication: *SHELXL97* ▶.

## Supplementary Material

Crystal structure: contains datablocks global, I. DOI: 10.1107/S160053681004585X/hb5725sup1.cif

Structure factors: contains datablocks I. DOI: 10.1107/S160053681004585X/hb5725Isup2.hkl

Additional supplementary materials:  crystallographic information; 3D view; checkCIF report

## Figures and Tables

**Table 1:** Hydrogen-bond geometry (Å, °) *Cg*1 is the centroidof the C3–C8 ring.

*D*—H⋯*A*	*D*—H	H⋯*A*	*D*⋯*A*	*D*—H⋯*A*
N1—H1⋯O3^i^	0.93	2.18	3.021 (2)	149
N1—H1⋯O4^i^	0.93	2.64	3.5217 (17)	158
N2—H2*A*⋯O3^i^	0.87	2.09	2.8855 (17)	152
N2—H2*B*⋯O4^ii^	0.92	2.14	3.0248 (19)	162
N4—H4*A*⋯O1^iii^	0.93	2.13	3.0273 (18)	161
N4—H4*B*⋯O2^iv^	0.96	1.90	2.8605 (16)	180
N5—H5*B*⋯O1^iv^	0.90	2.15	3.0440 (17)	170
O2—H2⋯O4^ii^	1.20	1.25	2.4500 (16)	173
C6—H6⋯*Cg*1^v^	0.93	3.10	3.676 (2)	122

Symmetry codes: (i) 
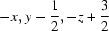
; (ii) 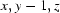; (iii) 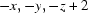; (iv) 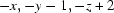; 
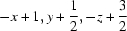
.

**Fig. 1.**
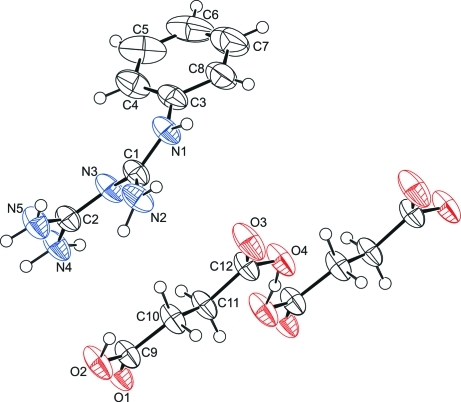
The molecular structure of (I) with displacement ellipsoids drawn at the 50% probability level.

**Fig. 2.**
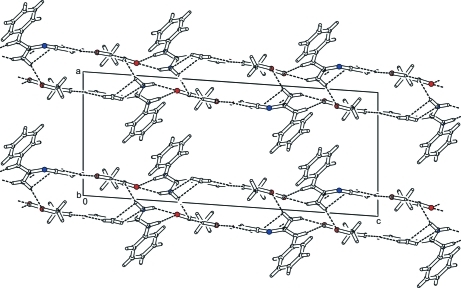
Part of the crystal structure of (I) showing formation of cation layers along [010].

**Fig. 3.**
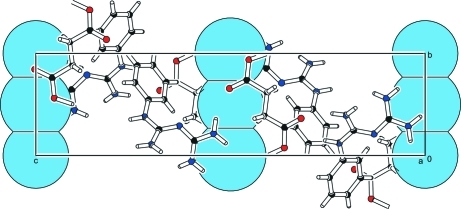
Part of the crystal structure of (I) with blue sphere filling the cavity.

**Fig. 4.**
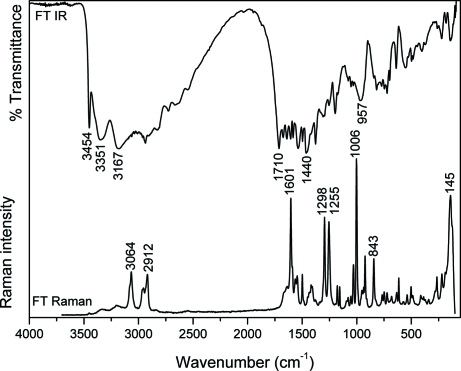
FTIR (compiled from nujol and fluorolube mulls) and FT Raman spectra of (I).

## supplementary crystallographic information

### Comment

Biguanides are strong σ- and π-donating ligands, which form stable complexes (Marchi *et al.*, 1999; Ray, 1961) with transition metal ions in high or unusual oxidation states. Biguanide complexes of boron have also been investigated as potential compounds for wood conservation (Anderson *et al.*, 1995).

Another application of biguanides lies in the field of medicine (Sirtori & Pasik, 1994; Clement & Girreser, 1999). *N*-dimethylbiguanide and *N*-phenylethylbiguanide are used for the treatment of diabetes mellitus (Thompson *et al.*, 1999; Ross *et al.*, 2004; Woo *et al.*, 1999), therapeutic treatment of pain, anxiety, memory disorders (Morain *et al.*, 1994). Biguanide and its derivatives are also produced as antimalarial drugs (Watkins *et al.*, 1987) and drugs with hypoglycaemic activity (Marchi *et al.*, 1999; Sirtori & Pasik, 1994; Shapiro *et al.*, 1959*a*,*b*).

We have prepared and discussed *N*-phenylbiguanide compounds within our project of searching for new materials with nonlinear optical properties (Matulková *et al.*, 2010; Matulková *et al.*, 2008), where *N*-phenylbiguanidinium(1+) cations can act as an polarizable compound with delocalized π-electron. The molecular conformation of title compound, (I), is illustrated in Fig. 1.

The hydrogen-bonding geometries in title compound are listed in list of hydrogen bonds and illustrated in Fig. 2. A number of intra- and intermolecular hydrogen bonds stabilize the molecular conformation. The crystal structure is built up chains (along the axis b) of hydrogen succinate anions with the shared hydrogen atoms with occupancy 0.5 (hydrogen bond O2 - H2···O4 with D···A distances of 2.451 (2) Å). These chains are interconnected by 2-phenylbiguanidium cations to form a three-dimensional network. A residue electron density of disordered molecules of methanol was found on the diferential Fourier map and the crystal structure was solved by a SQUEEZE method (van der Sluis & Spek, 1990). Free cavities of maximum on the Fourier map are indicated by blue spheres and are located in the 30% of crystal structure. The cavities can be filled by spheres of two types with the radii 2.247 Å and 2.076 Å (see Fig. 3). The unit cell contains two spheres of each size.

### Experimental

The crystals of the title compound, were obtained from solution of 0.2 g of *N*-phenylbiguanide (98%, Aldrich) and 0.14 g of succinic acid (p.a., Lachema) in 10 ml of water. The solution was left to crystallize at room temperature for several weeks. The colourless crystals obtained were filtered off, washed with methanol and dried in vacuum desiccator over KOH. The melting point ranges 410–412 K.

The infrared spectra were recorded at room temperature using DRIFTS and the nujol or fluorolube mull techniques on a Nicolet Magna 760 FTIR spectrometer with 2 cm^-1^ resolution (4 cm^-1^ resolution in FAR IR region) and Happ-Genzel apodization in the 85–4000 cm^-1^ region.

FTIR spectrum (cm^-1^): 3451 m; 3350 m; 3296 m; 3184 m; 2938 w; 2725 mb; 2660 mb; 2550 mb; 1712 m; 1674 m; 1640 *vs*; 1605 m; 1583 m; 1538 *vs*; 1497 s; 1455 m; 1429 m; 1419 m; 1334 w; 1310 w; 1294 w; 1256 m; 1198 m; 1176 m; 1074 w; 1054 wb; 1031 w; 957 mb; 842 w; 818 w; 804 w; 772 w; 747 m; 722 w; 698 m; 638 m; 579 m; 549 mb; 528 sh; 498 vw; 485 vw; 446 w; 414 vw; 367 wb; 261 mb; 221 w; 179 w; 143 w.

The Raman spectra of polycrystalline samples were recorded at room temperature on a Nicolet Magna 760 FTIR spectrometer equipped with the Nicolet Nexus FT Raman module (2 cm^-1^ resolution, Happ–Genzel apodization, 1064 nm Nd:YVO4 laser excitation, 450 mW power at the sample) in the 100–3700 cm^-1^ region.

FT Raman spectrum (cm^-1^): 3452 vw; 3335 vwb; 3200 vw; 3079 sh; 3066 m; 2966 m; 2953 m; 2918 m; 1668 w; 1639 w; 1604 *vs*; 1591 m; 1563 m; 1546 m; 1499 m; 1450 w; 1437 w; 1428 w; 1418 m; 1408 m; 1379 vw; 1341 vw; 1327 vw; 1295 s; 1255 s; 1178 m; 1156 m; 1095 w; 1079 w; 1054 w; 1030 m; 1002 *vs*; 952 w; 937 w; 923 m; 909 vw; 844 m; 767 w; 748 w; 722 w; 681 w; 633 w; 615 m; 572 vw; 538 w; 502 w; 488 w; 414 wb; 387 w; 374 w; 340 w; 283 w; 265 m; 220 m; 188 m; 141 *vs*; 124 m.

### Refinement

H atoms attached to C and N atoms were calculated in geometrically idealized positions, with C*sp*^3^ - H = 0.97 Å and C*sp*^2^ - H = 0.93 Å, and constrained to ride on their parent atoms, with *U*_iso_(H) = 1.5 *U*_eq_(C). The positions of H atoms attached to O and N atoms were localized in difference Fourier maps, and refined isotropically.

### Crystal data

**Table:**

C_8_H_12_N_5_^+^·C_4_H_5_O_4_^−^·CH_4_O	*F*(000) = 696
*M**_r_* = 327.35	*D*_x_ = 1.324 Mg m^−^^3^
Monoclinic, *P*2_1_/*c*	Mo *K*α radiation, λ = 0.71073 Å
Hall symbol: -P 2ybc	Cell parameters from 3693 reflections
*a* = 10.3280 (3) Å	θ = 1–27.1°
*b* = 6.4590 (1) Å	µ = 0.10 mm^−^^1^
*c* = 24.6770 (6) Å	*T* = 293 K
β = 94.0480 (13)°	Plate, colourless
*V* = 1642.06 (7) Å^3^	0.45 × 0.4 × 0.18 mm
*Z* = 4	

### Data collection

**Table:**

Nonius KappaCCD diffractometer	2613 reflections with *I* > 2σ(*I*)
Radiation source: fine-focus sealed tube	*R*_int_ = 0.030
horizontally mounted graphite crystal	θ_max_ = 27.1°, θ_min_ = 1.7°
Detector resolution: 9.091 pixels mm^-1^	*h* = −13→13
ω and π scans to fill the Ewald sphere	*k* = −8→8
18123 measured reflections	*l* = −31→31
3568 independent reflections	

### Refinement

**Table:**

Refinement on *F*^2^	Secondary atom site location: difference Fourier map
Least-squares matrix: full	Hydrogen site location: difference Fourier map
*R*[*F*^2^ > 2σ(*F*^2^)] = 0.056	H-atom parameters constrained
*wR*(*F*^2^) = 0.174	*w* = 1/[σ^2^(*F*_o_^2^) + (0.1087*P*)^2^ + 0.1338*P*] where *P* = (*F*_o_^2^ + 2*F*_c_^2^)/3
*S* = 1.07	(Δ/σ)_max_ = 0.001
3568 reflections	Δρ_max_ = 0.25 e Å^−^^3^
191 parameters	Δρ_min_ = −0.23 e Å^−^^3^
0 restraints	Extinction correction: *SHELXL97* (Sheldrick, 1997), Fc^*^=kFc[1+0.001xFc^2^λ^3^/sin(2θ)]^-1/4^
Primary atom site location: structure-invariant direct methods	Extinction coefficient: 0.055 (7)

### Special details

**Table:**

Geometry. All e.s.d.'s (except the e.s.d. in the dihedral angle between two l.s. planes) are estimated using the full covariance matrix. The cell e.s.d.'s are taken into account individually in the estimation of e.s.d.'s in distances, angles and torsion angles; correlations between e.s.d.'s in cell parameters are only used when they are defined by crystal symmetry. An approximate (isotropic) treatment of cell e.s.d.'s is used for estimating e.s.d.'s involving l.s. planes.
Refinement. Refinement of *F*^2^ against ALL reflections. The weighted *R*-factor *wR* and goodness of fit *S* are based on *F*^2^, conventional *R*-factors *R* are based on *F*, with *F* set to zero for negative *F*^2^. The threshold expression of *F*^2^ > σ(*F*^2^) is used only for calculating *R*-factors(gt) *etc*. and is not relevant to the choice of reflections for refinement. *R*-factors based on *F*^2^ are statistically about twice as large as those based on *F*, and *R*- factors based on ALL data will be even larger.

### Fractional atomic coordinates and isotropic or equivalent isotropic displacement parameters (Å^2^)

**Table:**

	*x*	*y*	*z*	*U*_iso_*/*U*_eq_	
N1	0.20697 (15)	−0.1226 (2)	0.77926 (5)	0.0515 (4)	
H1	0.1757	−0.1468	0.7435	0.062*	
N2	0.07708 (16)	−0.3948 (2)	0.79668 (5)	0.0570 (4)	
H2A	0.0599	−0.4106	0.7620	0.068*	
H2B	0.0280	−0.4630	0.8208	0.068*	
N3	0.18707 (15)	−0.2116 (2)	0.86872 (5)	0.0514 (4)	
N4	0.16449 (16)	−0.2862 (2)	0.95713 (5)	0.0537 (4)	
H4A	0.1524	−0.1453	0.9625	0.064*	
H4B	0.1638	−0.3785	0.9874	0.064*	
N5	0.18754 (16)	−0.5585 (2)	0.90047 (5)	0.0575 (4)	
H5A	0.2005	−0.6075	0.8702	0.069*	
H5B	0.1909	−0.6467	0.9288	0.069*	
C1	0.15804 (17)	−0.2481 (2)	0.81647 (6)	0.0464 (4)	
C2	0.17836 (16)	−0.3547 (2)	0.90737 (6)	0.0448 (4)	
O1	−0.16551 (14)	−0.15632 (17)	1.00242 (4)	0.0577 (4)	
O2	−0.16362 (15)	−0.43722 (16)	0.95237 (5)	0.0616 (4)	
H2	−0.1523	−0.4961	0.9068	0.074*	
O3	−0.10001 (17)	0.1366 (2)	0.81889 (5)	0.0738 (5)	
O4	−0.13315 (14)	0.42420 (17)	0.86173 (4)	0.0598 (4)	
C3	0.29311 (18)	0.0463 (2)	0.78891 (6)	0.0519 (4)	
C4	0.3920 (2)	0.0464 (3)	0.82884 (8)	0.0687 (6)	
H4	0.4044	−0.0663	0.8521	0.082*	
C5	0.4738 (3)	0.2177 (5)	0.83414 (11)	0.0929 (9)	
H5	0.5407	0.2191	0.8614	0.112*	
C6	0.4578 (3)	0.3832 (4)	0.80008 (14)	0.0964 (10)	
H6	0.5130	0.4967	0.8042	0.116*	
C7	0.3612 (3)	0.3805 (4)	0.76041 (14)	0.0911 (9)	
H7	0.3508	0.4928	0.7369	0.109*	
C8	0.2768 (2)	0.2135 (3)	0.75394 (9)	0.0694 (6)	
H8	0.2103	0.2139	0.7265	0.083*	
C9	−0.16134 (17)	−0.2385 (2)	0.95785 (6)	0.0464 (4)	
C10	−0.15527 (19)	−0.1185 (2)	0.90552 (6)	0.0488 (4)	
H10A	−0.0830	−0.1701	0.8864	0.059*	
H10B	−0.2341	−0.1447	0.8828	0.059*	
C11	−0.13977 (19)	0.1122 (2)	0.91303 (6)	0.0462 (4)	
H11A	−0.2155	0.1663	0.9293	0.055*	
H11B	−0.0647	0.1387	0.9379	0.055*	
C12	−0.12325 (17)	0.2254 (2)	0.86057 (6)	0.0462 (4)	

### Atomic displacement parameters (Å^2^)

**Table:**

	*U*^11^	*U*^22^	*U*^33^	*U*^12^	*U*^13^	*U*^23^
N1	0.0785 (10)	0.0498 (8)	0.0273 (6)	−0.0067 (6)	0.0109 (6)	0.0028 (5)
N2	0.0825 (11)	0.0601 (9)	0.0291 (7)	−0.0165 (7)	0.0088 (6)	−0.0007 (6)
N3	0.0828 (10)	0.0438 (7)	0.0288 (7)	−0.0077 (7)	0.0109 (6)	0.0018 (5)
N4	0.0905 (11)	0.0458 (8)	0.0258 (6)	−0.0013 (7)	0.0104 (6)	0.0035 (5)
N5	0.0926 (12)	0.0439 (8)	0.0368 (7)	0.0057 (7)	0.0099 (7)	0.0031 (6)
C1	0.0672 (10)	0.0430 (8)	0.0302 (7)	0.0002 (7)	0.0121 (7)	0.0014 (6)
C2	0.0593 (10)	0.0457 (8)	0.0300 (7)	−0.0025 (7)	0.0065 (6)	0.0021 (6)
O1	0.1019 (10)	0.0397 (6)	0.0324 (6)	−0.0008 (6)	0.0104 (6)	0.0029 (4)
O2	0.1145 (11)	0.0315 (6)	0.0402 (6)	−0.0014 (6)	0.0168 (6)	0.0058 (4)
O3	0.1439 (14)	0.0514 (7)	0.0279 (6)	−0.0011 (7)	0.0179 (7)	0.0000 (5)
O4	0.1054 (10)	0.0345 (6)	0.0410 (6)	−0.0023 (6)	0.0155 (6)	0.0084 (4)
C3	0.0733 (11)	0.0461 (9)	0.0391 (8)	−0.0031 (8)	0.0240 (8)	−0.0013 (6)
C4	0.0815 (14)	0.0792 (14)	0.0468 (10)	−0.0154 (10)	0.0140 (10)	0.0015 (9)
C5	0.0854 (16)	0.119 (2)	0.0775 (16)	−0.0400 (15)	0.0293 (13)	−0.0254 (15)
C6	0.105 (2)	0.0770 (16)	0.114 (2)	−0.0389 (14)	0.0597 (19)	−0.0213 (15)
C7	0.110 (2)	0.0545 (12)	0.116 (2)	−0.0070 (12)	0.0548 (19)	0.0143 (13)
C8	0.0849 (14)	0.0561 (11)	0.0711 (13)	0.0021 (9)	0.0319 (11)	0.0170 (9)
C9	0.0715 (11)	0.0329 (7)	0.0357 (8)	0.0001 (7)	0.0097 (7)	0.0041 (6)
C10	0.0817 (12)	0.0329 (8)	0.0325 (7)	−0.0001 (7)	0.0086 (7)	0.0027 (5)
C11	0.0779 (11)	0.0335 (7)	0.0281 (7)	−0.0031 (7)	0.0087 (7)	0.0026 (5)
C12	0.0741 (11)	0.0366 (8)	0.0281 (7)	−0.0040 (7)	0.0050 (7)	0.0029 (5)

### Geometric parameters (Å, °)

**Table:**

N1—C1	1.3495 (19)	C3—C4	1.368 (3)
N1—C3	1.417 (2)	C3—C8	1.385 (2)
N1—H1	0.9313	C4—C5	1.393 (3)
N2—C1	1.333 (2)	C4—H4	0.9300
N2—H2A	0.8679	C5—C6	1.363 (4)
N2—H2B	0.9206	C5—H5	0.9300
N3—C1	1.3242 (19)	C6—C7	1.348 (5)
N3—C2	1.3358 (19)	C6—H6	0.9300
N4—C2	1.3223 (18)	C7—C8	1.389 (4)
N4—H4A	0.9291	C7—H7	0.9300
N4—H4B	0.9561	C8—H8	0.9300
N5—C2	1.331 (2)	C9—C10	1.511 (2)
N5—H5A	0.8301	C10—C11	1.509 (2)
N5—H5B	0.9016	C10—H10A	0.9700
O1—C9	1.2245 (18)	C10—H10B	0.9700
O2—C9	1.2909 (19)	C11—C12	1.5069 (19)
O2—H2	1.2016	C11—H11A	0.9700
O3—C12	1.2164 (18)	C11—H11B	0.9700
O4—C12	1.2884 (19)		
			
C1—N1—C3	127.49 (14)	C4—C5—H5	119.4
C1—N1—H1	115.0	C7—C6—C5	119.3 (2)
C3—N1—H1	117.4	C7—C6—H6	120.3
C1—N2—H2A	121.6	C5—C6—H6	120.3
C1—N2—H2B	117.6	C6—C7—C8	121.3 (2)
H2A—N2—H2B	119.8	C6—C7—H7	119.4
C1—N3—C2	123.36 (14)	C8—C7—H7	119.4
C2—N4—H4A	119.0	C3—C8—C7	119.1 (2)
C2—N4—H4B	121.6	C3—C8—H8	120.4
H4A—N4—H4B	119.4	C7—C8—H8	120.4
C2—N5—H5A	120.7	O1—C9—O2	121.57 (13)
C2—N5—H5B	121.7	O1—C9—C10	123.48 (13)
H5A—N5—H5B	117.2	O2—C9—C10	114.95 (13)
N3—C1—N2	125.19 (14)	C11—C10—C9	114.32 (12)
N3—C1—N1	119.06 (15)	C11—C10—H10A	108.7
N2—C1—N1	115.64 (14)	C9—C10—H10A	108.7
N4—C2—N5	117.58 (14)	C11—C10—H10B	108.7
N4—C2—N3	116.65 (14)	C9—C10—H10B	108.7
N5—C2—N3	125.71 (13)	H10A—C10—H10B	107.6
C9—O2—H2	114.2	C12—C11—C10	113.04 (12)
C4—C3—C8	119.99 (19)	C12—C11—H11A	109.0
C4—C3—N1	123.32 (16)	C10—C11—H11A	109.0
C8—C3—N1	116.63 (18)	C12—C11—H11B	109.0
C3—C4—C5	119.0 (2)	C10—C11—H11B	109.0
C3—C4—H4	120.5	H11A—C11—H11B	107.8
C5—C4—H4	120.5	O3—C12—O4	120.62 (13)
C6—C5—C4	121.2 (3)	O3—C12—C11	122.59 (14)
C6—C5—H5	119.4	O4—C12—C11	116.78 (12)

### Hydrogen-bond geometry (Å, °)

**Table:**

Cg1 is the centroidof the C3–C8 ring.

**Table:**

*D*—H···*A*	*D*—H	H···*A*	*D*···*A*	*D*—H···*A*
N1—H1···O3^i^	0.93	2.18	3.021 (2)	149
N1—H1···O4^i^	0.93	2.64	3.5217 (17)	158
N2—H2A···O3^i^	0.87	2.09	2.8855 (17)	152
N2—H2B···O4^ii^	0.92	2.14	3.0248 (19)	162
N4—H4A···O1^iii^	0.93	2.13	3.0273 (18)	161
N4—H4B···O2^iv^	0.96	1.90	2.8605 (16)	180
N5—H5B···O1^iv^	0.90	2.15	3.0440 (17)	170
O2—H2···O4^ii^	1.20	1.25	2.4500 (16)	173
C6—H6···Cg1^v^	0.93	3.10	3.676 (2)	122

Symmetry codes: (i) −*x*, *y*−1/2, −*z*+3/2; (ii) *x*, *y*−1, *z*; (iii) −*x*, −*y*, −*z*+2; (iv) −*x*, −*y*−1, −*z*+2; (v) −*x*+1, *y*+1/2, −*z*+3/2.
